# 3DFlex: determining structure and motion of flexible proteins from cryo-EM

**DOI:** 10.1038/s41592-023-01853-8

**Published:** 2023-05-11

**Authors:** Ali Punjani, David J. Fleet

**Affiliations:** 1 Department of Computer Science, University of Toronto, Toronto, Ontario Canada; 2 Vector Institute for Artificial Intelligence, Toronto, Ontario Canada; 3 Structura Biotechnology Inc., Toronto, Ontario Canada; 4 Google Research, Toronto, Ontario Canada

**Keywords:** Cryoelectron microscopy, Software, Machine learning, Image processing

## Abstract

Modeling flexible macromolecules is one of the foremost challenges in single-particle cryogenic-electron microscopy (cryo-EM), with the potential to illuminate fundamental questions in structural biology. We introduce Three-Dimensional Flexible Refinement (3DFlex), a motion-based neural network model for continuous molecular heterogeneity for cryo-EM data. 3DFlex exploits knowledge that conformational variability of a protein is often the result of physical processes that transport density over space and tend to preserve local geometry. From two-dimensional image data, 3DFlex enables the determination of high-resolution 3D density, and provides an explicit model of a flexible protein’s motion over its conformational landscape. Experimentally, for large molecular machines (tri-snRNP spliceosome complex, translocating ribosome) and small flexible proteins (TRPV1 ion channel, *αVβ*8 integrin, SARS-CoV-2 spike), 3DFlex learns nonrigid molecular motions while resolving details of moving secondary structure elements. 3DFlex can improve 3D density resolution beyond the limits of existing methods because particle images contribute coherent signal over the conformational landscape.

## Abstract (web summary)

3D Flexible Refinement (3DFlex) is a generative neural network model for continuous molecular heterogeneity for cryo-EM data that can be used to determine the structure and motion of flexible biomolecules. It enables visualization of nonrigid motion and improves 3D structure resolution by aggregating information from particle images spanning the conformational landscape of the target molecule.

## Main

Proteins form the molecular machinery of the cell. They are inherently dynamic, often exhibiting a continuous landscape of conformations, with motion tightly linked to function. Methods that uncover protein motion and the conformational landscape have the potential to illuminate fundamental questions in structural biology, and to enhance the ability to design therapeutic molecules that elicit specific functional changes in a target protein.

Single-particle cryo-EM collects thousands of static two-dimensional (2D) particle images that, in aggregate, may span the target protein’s 3D conformational space. Cryo-EM therefore holds great promise for uncovering both the atomic-resolution structure and motion of biologically functional moving parts^1^. This highlights the need for methods for resolving continuous motion and structure from static 2D images. Local reconstruction via multi-body refinement^2,3^ is effective for macromolecules with sufficiently large, rigid subunits, given masks to isolate the subunits. Principal component analysis^4^ and linear subspace methods, such as 3D variability analysis (3DVA)^5^ approximate a particle’s space of conformations as a weighted sum of basis density maps. Nonlinear manifold embedding methods^6,7^, including deep-learning models such as CryoDRGN^8^, offer even more expressive power. Such density-based methods are widely applicable, handling compositional and conformational heterogeneity; they provide encouraging evidence that one can estimate structural variation from a single heterogeneous dataset. However, the existing methods have limitations. Local and multi-body refinements are not readily applicable to highly flexible motion or the motion of small subunits (SSUs). Linear subspace models are limited to relatively simple, small motions. Density-based models do not explicitly estimate motion, and as a consequence are not able to aggregate signal across a particle’s conformational landscape to improve the resolution of 3D density in flexible regions.

The development of a computational method that can uncover both fine structural detail and nonrigid protein motion in the presence of continuous flexibility must overcome multiple challenges. It entails joint optimization of many unknowns, including the 3D structure of the density map and a representation of the position of each particle image on the conformational landscape of the protein. It is also unclear how to aggregate signal across all particles, attenuating noise and improving map quality with sufficient regularization to avoid overfitting. Recent methods that do estimate motion, such as e2gmm (ref. ^9^) and hypermolecules^10^, address some but not all of these challenges.

We introduce 3D Flexible Refinement (3DFlex), a deep neural network model of continuously flexible protein molecules. 3DFlex is a motion-based heterogeneity model that directly exploits the knowledge that most conformational variability of a protein is a result of physical processes that tend to transport density, preserving local geometry (for example, the relative positions and/or orientations of side chains). We formulate 3DFlex as a generative deep-learning architecture that captures conformational variability in terms of a single high-resolution ‘canonical’ 3D density map of the molecule, and a parameterized latent space of deformation fields encoding flexible (nonrigid) motion. The motion model is used to deform the canonical density via convection, yielding all conformations captured by the model. In 3DFlex, the latent coordinates of each particle image, the deformation field generator and the canonical density are jointly learned from image data using a specialized training algorithm, with minimal previous knowledge about the flexibility of the molecule.

Results on experimental cryo-EM data show that 3DFlex addresses the challenges of uncovering structure and motion of flexible proteins. On a dataset of tri-snRNP spliceosome particles^11^, 3DFlex learns a wide range of nonrigid motions, including subunits bending across a span of more than 20 Å. In doing so, the algorithm aggregates structural information from all conformations into a single, optimized density map that resolves high-resolution details in α-helices and β-sheets even in the flexible domains. It estimates motion with sufficient precision to improve the resolution of small flexible parts that are otherwise poorly resolved in conventional and local, focused refinements. We demonstrate this ability with a dataset of TRPV1 ion-channel particles^12^, where 3DFlex improves the resolution of peripheral α-helices in the flexible soluble domains. Additional experiments on a SARS-CoV-2 spike protein^13^, an *αVβ*8 integrin^14^ and a translocating ribosome^15^ demonstrate that 3DFlex can reveal structures not resolved by conventional refinement, and can map out conformational variations. With such capabilities, 3DFlex opens up new avenues of inquiry into the study of biological mechanisms and function involving motion.

## Results

### 3DFlex

3DFlex is a generative neural network method that determines the structure and motion of flexible biomolecules from cryo-EM images. Central to 3DFlex is the assumption that conformations of a dynamic protein are related to each other through deformation of a single 3D structure. Specifically, a flexible molecule is represented in terms of (1) a canonical 3D map, (2) latent coordinate vectors that specify positions over the protein’s conformational landscape and (3) a flow generator that converts a latent coordinate vector into a deformation field that convects the canonical map into the corresponding conformation. The canonical 3D map, the flow generator, and a latent coordinate vector for each particle image are jointly learned from experimental data.

**Fig. 1:**
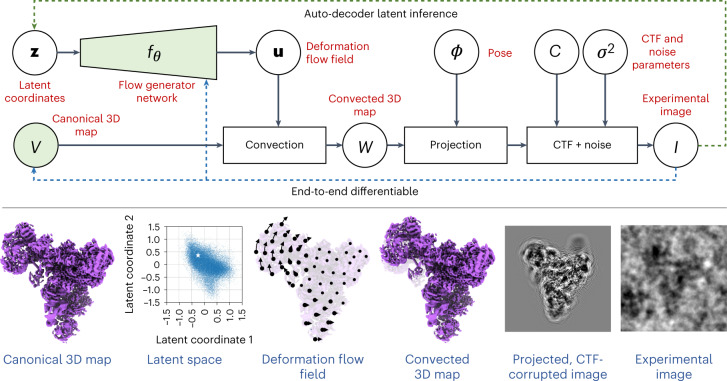
The 3DFlex model represents the flexible 3D structure of a protein as deformations of a single **canonical** 3D density map *V*. Under the model, a single-particle image is associated with low-dimensional latent coordinates that encode the conformation for the particle in the image. A neural flow generator network *f*_*θ*_ converts the latent coordinates into the flow field **u** and a convection operator then deforms the canonical density to generate a convected map *W*. This map can then be projected along the particle viewing direction determined by the pose *ϕ*, CTF corrupted and compared against the experimental image.

Under the 3DFlex model (Fig. 1), a single-particle 2D image *I*_*i*_ is generated as follows. First, a *K*-dimensional latent coordinate vector, **z**_*i*_, is input to a flow generator, *f*_*θ*_(**z**_*i*_), with parameters *θ*. The generator produces a 3D deformation field that is used to convect the canonical 3D density map, *V*. The convected density, denoted *D*(*f*_*θ*_(**z**_*i*_), *V*), is then projected to 2D, contrast transfer function (CTF) modulated and corrupted by additive noise *η*; that is,

1 $${I}_{i}={C}_{i}\,P({\phi }_{i})\,D({f}_{\theta }({{{{\bf{z}}}}}_{i}),V)\,+\,\eta ,$$

where *C*_*i*_ is the CTF operator and *P*(*ϕ*_*i*_) is the projection operator for pose *ϕ*_*i*_ (the rigid coordinate transform between the microscope and the canonical map). Fitting 3DFlex to experimental images entails optimizing the flow generator parameters *θ*, the canonical map *V* and the latent coordinates **z**_*i*_, to minimize the data log likelihood under the probabilistic model (equation (1)). For the current development of 3DFlex, we use a white noise model and assume poses *ϕ*_*i*_ and CTF parameters are known, for example, from a standard cryo-EM refinement algorithm, although these parameters could also be reoptimized by 3DFlex.

Computationally determining structure and motion from noisy cryo-EM data is challenging. Hence, there are several important design choices that define an effective model architecture and optimization procedure. Briefly (see Methods for details), we represent and optimize the canonical density *V* as a real-space occupancy grid. The flow generator network is a multi-layer perceptron (MLP) that outputs a flow vector at each vertex of a tetrahedral mesh. Interpolation within mesh elements, using finite-element methods, yields a flow field that maps density from canonical coordinates to the observed coordinate frame of a given particle image. Thus, 3DFlex conserves density, as do conventional, rigid reconstruction methods. Further, central to the optimization is a regularizer that encourages locally smooth and rigid motion in regions of the canonical map with high density.

After specifying the resolution and topology of the tetrahedral mesh, and the number of layers and hidden units of the MLP, the latent coordinates for each particle image can be initialized randomly or set to coordinates provided by another method such as 3DVA (ref. ^5^). During learning, one can optimize the canonical map, the MLP weights and the per-particle latent coordinates simultaneously, or in an alternating form of block coordinate descent. To help encourage a smooth latent representation, we also regularize the latent coordinates by injecting uncertainty into the latent positions at each iteration of network weight updates. Learning is performed using low-resolution particle images to reduce computational cost, and also to enable half-set Fourier shell correlation (FSC) validation of final reconstructions at full resolution (Methods).

Once the parameters of the flow generator and the latent coordinates of the particle images have been learned, we perform high-resolution refinement of the canonical map. The goal is to exploit the improved nonrigid alignment provided by the flow generator to resolve fine-grained detail in the canonical map. To that end, given the fixed flow generator and latent coordinates, we use L-BFGS^16^ to optimize the canonical map against particle data, now at full resolution, using a conventional least-squares objective. Further details of model architecture, design choices and optimization are provided in the Methods.

### Datasets and experimental details

We apply 3DFlex to five experimental cryo-EM datasets: a tri-snRNP spliceosome^11^, a TRPV1 ion channel^12^, a SARS-CoV-2 spike protein^13^, an *αVβ*8 integrin^14^ and a translocating ribosome^15^. (Quantitative analyses with synthetic data are reported in Supplementary Material.) For each dataset, we first compute a rigid consensus refinement using all particle images. Nonuniform refinement^17^ is used to provide good (rigid) alignments despite conformational heterogeneity in the data. The resulting poses *ϕ*_*i*_ are fixed, and particle images are downsampled to a limited resolution during training of the 3DFlex model. 3DFlex is run with a real-space mask that excludes solvent in the canonical density *V*. In the case of membrane proteins, a separate mask is used to enforce zero deformation in the region of detergent micelle or lipid nanodisc.

Unless otherwise stated, model parameters are set to default values (Methods). The default architecture for the flow generator is a six-layer MLP with 64 hidden units per layer. Its weights are initialized to random values. A regular tetrahedral mesh is automatically generated to cover the spatial extent of the consensus refinement. Optionally, the user may adjust the mesh topology (Methods). Beyond the structure and topology of the mesh, no previous information is provided about the specific form of heterogeneity in each dataset. Once 3DFlex is trained, the final high-resolution refinement step yields two half-maps from which FSC can be used to measure improvements in global or local resolution. Each experiment is run on a NVIDIA Tesla V100 GPU with 32 GB of video RAM, typically requiring 10 to 20 h.

For visualization of the canonical density, the half-maps from 3DFlex are combined, filtered by their FSC curve and *B*-factor sharpened. Where noted, resulting maps are locally filtered to aid in visualization. To display conformational changes in figures, we select points in the 3DFlex latent space, for example, **z**_display_, and then generate the corresponding convected densities, *W*_display_ = *D*(*f*_*θ*_(**z**_display_), *V*). These densities are rendered overlaid in multiple colors, and with reference position guide markers to help visualize the motion. Supplementary Videos depict structure and motion with greater clarity.

### snRNP spliceosome: large nonrigid deformations of a molecular machine

The U4/U6.U5 tri-snRNP complex represents a large part of the spliceosome, with several moving parts, linkages and flexible domains^11^. The dataset comprises 138,899 particle images (EMPIAR-10073), with box size of 380 pixels of width 1.4 Å. They are first processed through heterogeneous refinement in cryoSPARC (ref. ^18^) to remove particles missing the ‘head’ region, yielding 102,500 final particles. These are downsampled to a box size of 180 pixels (2.95 Å wide) for training 3DFlex. For the snRNP complex we use a five-dimensional latent space; a larger latent space enables 3DFlex to discover more distinct, intricate motions. A regular tetrahedral mesh with 1,601 vertices and 5,859 cells (approximately 18 Å wide) is automatically generated to cover the input (consensus) map. Given the latent coordinates and flow generator learned by 3DFlex, the full-resolution particle images are used to reconstruct the canonical density (in separate half-maps) using L-BFGS. Total training time is 18 hours.

**Fig. 2:**
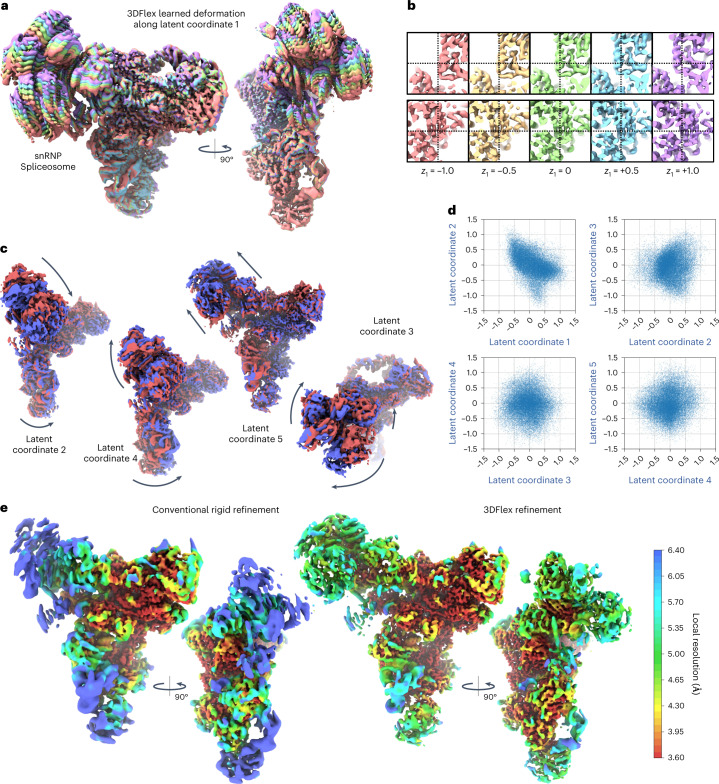
3DFlex with a 5D latent space applied to particle images of an snRNP Spliceosome complex, demonstrating the capacity for 3DFlex to resolve multiple modes of nonrigid deformation simultaneously, while also capturing high-resolution structural detail. See Supplementary Video 1. **a**, Colored series of convected densities learned by 3DFlex, at five positions along the first latent dimension, ranging from minus one to plus one standard deviation. **b**, Same as **a** but focused on key structural details in the head region of the protein. The top row shows an α-helix that translates several Angstroms. The bottom row shows a β-sheet, which translates and deforms. **c**, Convected densities from 3DFlex at minus one (red) and plus one (blue) standard deviations in the latent space, along each of the remaining four latent dimensions. Each dimension resolves a different type of motion within the same model. **d**, Scatter plots showing the final distribution of learned particle latent coordinates across the dataset. **e**, The left shows a density map from conventional nonuniform rigid refinement colored by local resolution. The right shows a canonical density map from 3DFlex, colored on the same local resolution color scale. The two maps are filtered by local resolution to aid in visualizing weak density in low-resolution areas in the conventional refinement.

3DFlex recovers five dimensions of motion (Fig. 2 and Supplementary Video 1), each of which captures a different type of bending or twisting in the molecule. There are two large moving parts, the head and foot, attached to the more rigid central body. In the learned deformation fields, the foot region largely moves as a rigid subpart, with a hinge-like linkage to the body. The head region exhibits large motion and substantial internal flexibility, aspects of which are encoded in each of the latent directions.

While recovering 3D motion, 3DFlex also determines high-resolution detail in the canonical map. This occurs through the aggregation of signal across the conformational landscape, with nonrigid alignment between particle images and the canonical map. Individual *α*-helices can be seen translating several Angstroms while retaining side-chain features. Likewise, a β-sheet in the flexible head region is resolved with separated β-strands, despite the nonrigid motion present. Because the flow generator was trained on downsampled images (pixel size 2.95 Å, and hence a Nyquist limit of 5.9 Å), these structural features represent additional signal that is resolved from the original data as a consequence of the accuracy of the recovered motion (that is, via nonrigid alignment).

In regions of substantial motion and flexibility, differences between a static conventional refinement and 3DFlex are dramatic (Fig. 2e); for example, local resolution in the center of the head region is improved from 5.7 to 3.8 Å. For a complex as large as the snRNP, it is worth noting that one could create manual masks around regions that are expected to be rigid, and then perform local or multi-body refinement^3^. Such refinement techniques can improve resolution and map quality in domains such as the foot, which remains rigid despite motion relative to the remainder of the molecule. In contrast, 3DFlex does not require manual masking or previous knowledge about the motion of the molecule. It can detect and then correct for nonrigid flexibility across the entire molecule, including the head, which is considerably less rigid than the foot.

### TRPV1 ion channel: capturing flexible motion improves resolution

The TRPV1 ion channel is a 380 kDa tetrameric membrane protein that acts as a heat- and capsaicin-activated sensory cation channel^12^. We process 200,000 particles of TRPV1 in nanodisc (EMPIAR-10059), downsampled to a box size of 128 pixels of width 1.21 Å for training 3DFlex. A tetrahedral mesh with 1,054 vertices and 3,892 cells (about 14 Å wide) is generated to cover the density. Based on 3DVA (ref. ^5^), it is evident that TRPV1 exhibits smooth, relatively small deformations. As such, a smaller architecture is sufficient, and helps to mitigate the risk of overfitting; we use a three-layer MLP with 32 units per layer for the flow generator and a 2D latent space. We initialize the latent coordinates to those from 3DVA, which helps avoid poor local minima that can be problematic with small proteins. Once 3DFlex is trained, the original particles (pixel size 1.21 Å) are used to reconstruct the canonical density (in separate half-maps) to high resolution. Symmetry is not enforced during training but the half-maps are symmetrized post hoc to C4 to enable comparison with the rigid refinement baseline (Methods). During training, the micelle region is set to have zero deformation.

**Fig. 3:**
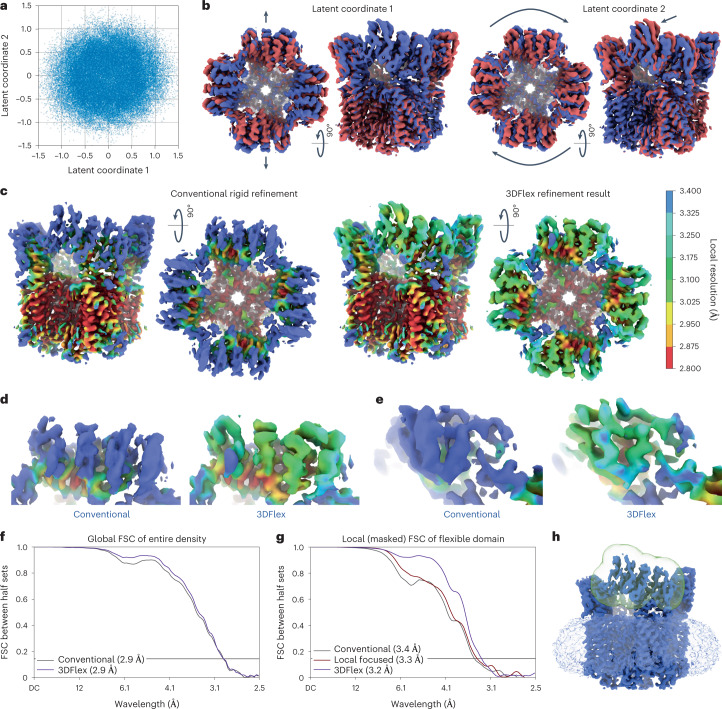
3DFlex applied to the TRPV1 dataset, demonstrating the capacity for 3DFlex to resolve motion of smaller, membrane proteins. See Supplementary Videos 2 and 3. **a**, Scatter plots showing the distribution of learned particle latent coordinates. **b**, Convected densities from 3DFlex at minus one (red) and plus one (blue) standard deviations along each of the two dimensions in the latent space. The first reveals a motion where opposite soluble domains move together or apart. The second reveals motion of all four soluble domains twisting around the axis of the central pore. **c**, Density maps from conventional nonuniform refinement (left) and the 3DFlex canonical map, both colored by local resolution on the same scale. Both are filtered and sharpened identically, and displayed at the same threshold level. **d**,**e**, Detailed views from top (**d**) and side (**e**) showing helical density in the flexible soluble domains. **f**,**g**, FSC curves are measured between half-map reconstructions from disjoint half-sets of particles and during training experimental data only up to 4.3 Å resolution is used (DC denotes constant signal). **f**, FSC of the entire map. **g**,**h**, FSC of the flexible peripheral domain (mask shown in **h**) including FSC curve (red) for local rigid refinement using the same mask (**g**).

The resulting 3DFlex model captures two types of flexible, coordinated motion among the four peripheral soluble domains of the ion channel (Fig. 3 and Supplementary Video 2). Along the first latent dimension, each pair of opposing subunits bends toward each other while the other pair bends apart. The second involves all four subunits twisting concentrically around the channel’s pore axis. In both cases, the peripheral-most helices move by approximately 6 Å. Both motions are nonrigid and involve flexure of substantial regions of the protein density.

In a conventional refinement, these motions are detrimental to reconstruction quality and resolution (Fig. 3c and Supplementary Video 3). Several *α*-helices in the soluble region are so poorly resolved that helical pitch is barely visible. Local resolution reaches 2.8 Å in the rigid core of the channel, but only 4 Å at the periphery. 3DFlex, on the other hand, estimates the motion of these domains, improving local alignment, which yields better resolution and map quality. During training of the flow generator, 3DFlex only makes use of downsampled images with a pixel size of 2.15 Å (a maximum Nyquist wavelength of 4.3 Å). But with improved alignment at full resolution (pixel size 1.21 Å), gold-standard FSC and local resolution measurements using the two half-set reconstruction in 3DFlex show that it recovers consistent structural information well beyond 4.3 Å. Local resolutions in peripheral helices improve to 3.2 Å revealing helical pitch and side-chain details. The separate half-set reconstructions from 3DFlex allow us to use established validation procedures to measure the improvement derived from nonrigid motion estimation. The FSC curve for the entire density (Fig. 3f) improves slightly in 3DFlex compared to conventional refinement. This indicates that in the rigid core of the molecule, 3DFlex has not lost structural information. To investigate the effect in the peripheral domains, we construct a soft-edged mask around one of the flexible domains (Fig. 3h) and test the mask for tightness using noise substitution^19^. FSC curves within this mask (Fig. 3g) show that 3DFlex improves the average resolution from 3.4 to 3.2 Å as well as increasing the signal-to-noise ratio at low and medium resolutions. This improvement means that 3DFlex has resolved more structural information than conventional refinement, and confirms that the nonrigid motion learned by 3DFlex is a better model of the particle than the rigid model.

3DFlex improves the reconstruction of TRPV1 by explicitly modeling nonrigid deformation. As a baseline, we also perform a local focused refinement using symmetry-expanded particles and the same mask (Fig. 3h) to isolate a soluble domain. Local refinement does not improve the density or resolution of the domain beyond conventional refinement (Fig. 3g), as expected since each soluble domain is less than 50 kDa and deforms flexibly. We believe that this comparison illustrates an additional advantage of 3DFlex. Unlike local and multi-body refinement methods that assume rigidity and attempt to fit separate pose parameters for each masked region, 3DFlex exploits correlations between moving parts, making it possible to infer the position of all parts, even though individually each is too small to align reliably. In the case of TRPV1, the four soluble domains deform in different directions by different amounts, but 3DFlex infers their positions in a given image jointly.

### SARS-CoV-2 spike protein in prefusion state

The SARS-CoV-2 spike protein^13^ is a natural candidate for 3DFlex as it exhibits continuous flexibility that cannot be resolved by standard classification techniques, especially in the neighborhoods of the receptor binding domain (RBD) and N-terminal domain (NTD). We process 2,139 raw cryo-EM movies (EMPIAR-10516) in cryoSPARC to obtain 113,511 particle images (box size 256; pixel size 1.396 Å). After downsampling (box size 140; pixel size 2.55 Å), we train 3DFlex with a 3D latent space.

**Extended Data Fig. 1:**
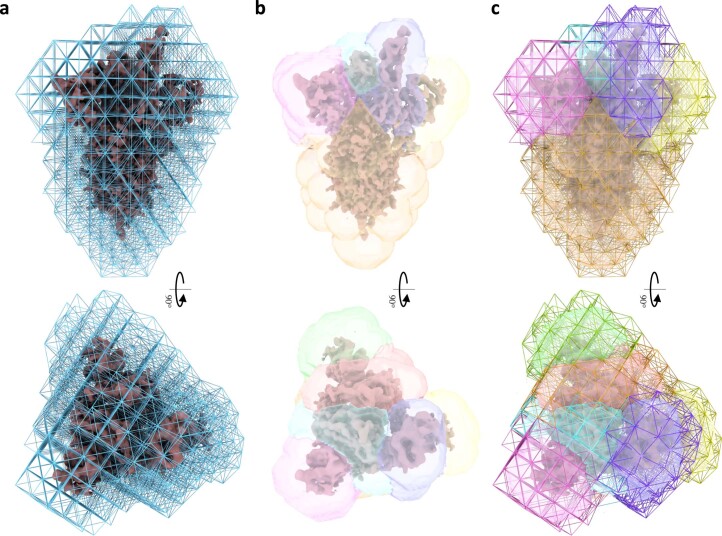
Mesh Topologies. Examples of mesh topology that can provided to 3DFlex for the SARS-CoV-2 spike protein (see Fig. 4). **a**: a regular mesh, with all mesh elements connected to their neighbors. This does not allow adjacent protein domains to easily separate or move in different directions. **b**: coarse separation of domains. **c**: an irregular mesh constructed by fusing sub-meshes for each domain at the interfaces where density is to be continuous. 3DFlex still learns motion at all mesh nodes jointly and from scratch, but is now able to model adjacent domains that move in different directions easily.

By default, 3DFlex uses a regular tetrahedral mesh, but the method works with any mesh geometry. As discussed in Methods, the mesh topology can be adjusted to introduce additional inductive bias. This is useful for resolving motion of adjacent domains that move differently from each other. For the spike protein we obtained good results with a mesh constructed using a submesh for each RBD and NTD domain, fused to a submesh for the central trimer of S2 domains (Extended Data Fig. 1). We provided coarse boundaries between adjacent RBD and NTD domains from which the submeshes and complete mesh were automatically constructed (Methods). The mesh element size is 14 Å. A custom mesh topology provides helpful inductive bias but does not provide 3DFlex with information about the direction nor types of molecular motion present in the data. Rather, 3DFlex must still learn nonrigid deformations from scratch across all mesh nodes jointly during training.

**Fig. 4:**
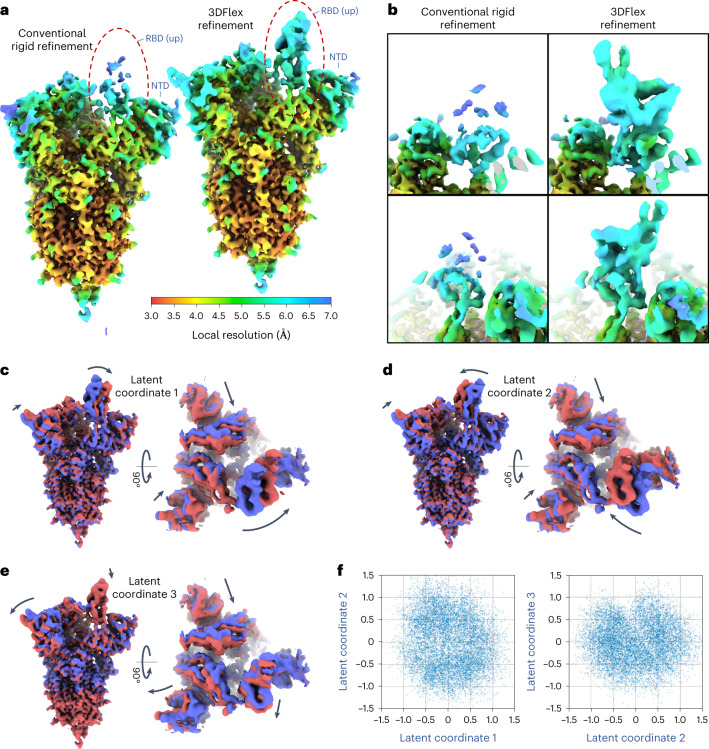
3DFlex applied to the SARS-CoV-2 spike protein. See Supplementary Video 4. **a**, The left shows a density map from conventional nonuniform refinement colored by local resolution. The right shows a canonical density map from 3DFlex with the same local resolution color scale. Both maps are filtered by local resolution, sharpened identically and displayed at the same threshold level to simplify visual comparison of map quality. **b**, Enlarged region of the map to show the detailed structure of the up-RBD that is resolved by 3DFlex but not by the conventional refinement. **c**–**f**, Convected densities from 3DFlex at minus one (red) and plus one (blue) standard deviations in the latent space (**f**) along coordinate axes for dimensions 1 (**c**), 2 (**d**), and 3 (**e**).

The resulting 3DFlex model captures multiple coordinated bending motions of the RBDs and NTDs in the S1 region of the spike protein (Fig. 4c–e and Supplementary Video 4). The up-RBD shows the largest motions, as expected. In conventional refinement, the up-RBD is essentially unresolved and appears as broken and/or blurred density, while with 3DFlex refinement, the up-RBD is intact and resolved at a resolution around 5 Å (Fig. 4ab). This is notable as the up-RBD is the functionally active part of the spike in prefusion state. There also appears to be structure in the latent landscape (Fig. 4f) suggesting that certain positions of the RBDs and NTDs are more energetically favorable than others, although we do not analyze this landscape in detail here.

### *αVβ*8 Integrin with two Fabs

The *αVβ*8 integrin is a highly flexible protein involved in cell differentiation during development, and in fibroinflammatory processes and antitumor immunity^14^. We process 84,266 particles (EMPIAR-10345) of *αVβ*8 integrin with two Fabs bound. 3DFlex is trained on downsampled images (pixel size 3.15 versus 1.345 Å), with a 2D latent space and a regular tetrahedral mesh with 477 vertices and 1452 cells (22 Å wide).

**Fig. 5:**
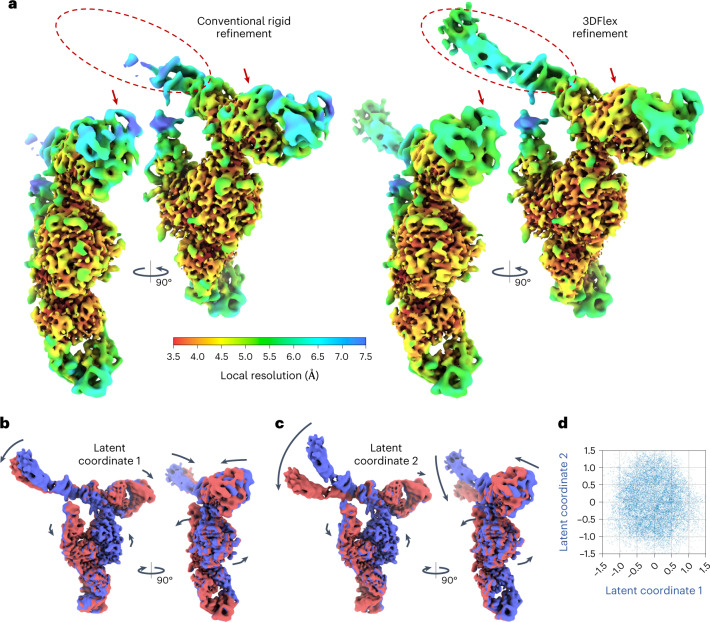
3DFlex on the *αVβ*8 integrin. See Supplementary Video 5. **a**, The left shows a density map from conventional nonuniform refinement colored by local resolution. The right shows a canonical density map from 3DFlex with the same local resolution color scale. Both maps are locally filtered, sharpened identically and displayed at the same threshold level to simplify visual comparison of map quality. 3DFlex exhibits clear improvement in map structure in regions indicated in the vicinity of the flexible arm of the protein (dashed outline) and in flexible Fabs (red markers) **b–d,** Convected densities from 3DFlex at minus one (red) and plus one (blue) standard deviations in the latent space (**d**) along coordinate axes for dimensions 1 (**b**) and 2 (**c**). The motion of the arm is captured mainly by the second latent dimension.

3DFlex resolves large motions of the flexible arm of the integrin (Fig. 5b,c and Supplementary Video 5). In doing so, 3DFlex enables improved reconstruction of the highly flexible region that is barely resolved by conventional refinement; local resolution improves from 8+ to 6.5 Å (Fig. 5a). Continuous motion of this magnitude (larger than the width of the arm) is not well modeled by simpler continuous heterogeneity techniques such as 3DVA (ref. ^5^). 3DFlex uncovers joint bending and motion of the flexible arm as well as the core region of the protein and the two Fabs. The local resolution in the core region is also slightly improved by 3DFlex, from 3.6 to 3.5 Å, and local resolutions in other regions (red markers in Fig. 5a) also improve.

### Translocating ribosome with elongation factor G

Ribosomal translocation involves coordinated motion of multiple subunits of the ribosome with participation of elongation factor G (EF-G), a translational GTPase^15^. To demonstrate the capacity of 3DFlex to model complex, nonrigid motions of a molecular machine undergoing a reaction, we process a dataset of 58,433 particle images (box size 288; pixel size 1.16 Å) of the Ribosome-EF-G complex in the presence of GDP (EMPIAR-10792). The original study^15^ separated major conformational states using 3D Classification. We combine particles classified into Hybrid(GDP+Pi), Hybrid(GDP) and Chimeric(GDP) states^15^ into a single-particle set, discarding the original labels. These images are downsampled (pixel size 2.38 Å), and used to train 3DFlex with a 2D latent space. We use a custom mesh topology, specified by coarse boundaries between the large subunit, SSU, EF-G and transfer RNAs subunits, from which submeshes and a complete fused mesh are automatically generated (Methods). The final mesh, with element size 14 Å, covers the entire Ribosome-EF-G complex.

**Fig. 6:**
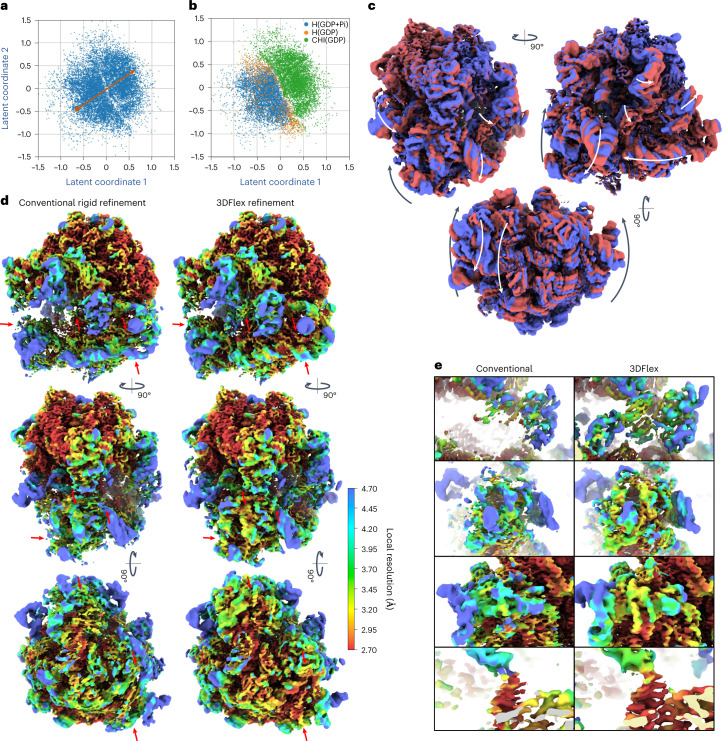
3DFlex applied to a translocating ribosome. See Supplementary Video 6. **a**, The latent distribution of particles modeled by 3DFlex, with the orange line traversing the two major conformations. **b**, The same distribution colored by labels from discrete classification results of ref. ^15^ showing three identified states. **c**, Front, side and bottom views of convected densities from 3DFlex at minus one (red) and plus one (blue) standard deviations along the orange line in (**a**). **d**, The left shows the side, front and bottom views of density map from conventional nonuniform refinement colored by local resolution. The right shows a canonical density map from 3DFlex with the same local resolution color scale. Both maps are locally filtered, sharpened identically and displayed at the same threshold level to simplify visual comparison of map quality. 3DFlex exhibits improvement in map structure, with comparison aided by red markers. **e**, The same as (**a**) but enlarged views of (top to bottom) EF-G, head region, back of SSU and tRNA.

3DFlex learns highly coordinated, intricate motion of multiple parts (Fig. 6c and Supplementary Video 6). In the learned model, the ribosome undergoes a transition between two major conformations with the EF-G causing a swiveling of the SSU and head region and displacement of the tRNAs, as well as smaller conformational changes within each of the major states. The flow generator captures the simultaneous motion of the SSU, head region, EF-G, tRNAs and peripheral RNA helices. The distribution of latent coordinates (Fig. 6a) shows a separation of particles between the two major conformations, which correspond to the Hybrid(GDP+Pi) and Chimeric(GDP) states that were originally separated by 3D classification^15^ (Fig. 6b). We also find that 3DFlex delineates the conformational change between the major Hybrid(GDP+Pi) state and the less-populated intermediate Hybrid(GDP) state, although we do not analyze the distribution in detail here. These results demonstrate the use of 3DFlex for mapping functional motion of complex cellular machinery, in cases where the dataset contains particles that span multiple states along the reaction.

Despite the large motions present, 3DFlex refinement recovers high-resolution detail even in regions that are blurred or missing in conventional refinement (Fig. 6d). This includes RNA helices in the SSU (resolved at local resolution of 3 Å), α-helices in the head region and tRNA density (Fig. 6e).

## Discussion

3DFlex complements existing reconstruction methods for heterogeneous data. 3D Classification^20–23^ can approximate continuous heterogeneity by partitioning the input particles and computing a rigid reconstruction on individual clusters. Recent methods enable larger numbers of classes^7^, mitigating potential problems caused by conformational diversity within each class, but they also require increasingly large datasets. 3DFlex is data efficient, as every particle contributes to the canonical density, regardless of its conformational state.

Local, focused refinement^20,22^ and multi-body refinement^2^ methods allow high-resolution refinement of flexible molecules by assuming the molecules are composed of a small number of rigid parts. Masks are needed to separate the parts, and each part must have sufficient molecular weight (typically 150 kDa or more) for accurate rigid alignment^3^. 3DFlex recovers motion and structure of nonrigidly deformable parts across an entire molecule, with control over the complexity of motion via mesh granularity and smoothness regularizers. Default mesh generation is automatic, but one also has the option to adjust mesh connectivity by separating subunits and domains to better resolve boundaries.

Techniques such as normal-modes analysis^24^ make assumptions about the local energy landscape of a protein around a base state of a molecule to predict flexibility. Methods have been proposed to exploit such models to recover improved density maps from cryo-EM data of flexible molecules^25^. 3DFlex does not presuppose knowledge of the energy landscape or dynamics of the molecule, but rather learns this from the image data.

Density-based methods have recently emerged to learn continuous heterogeneity. Eigen-methods model the space of 3D conformations as a linear subspace^4,26,27^; notably, 3DVA (ref. ^5^) computes and visualizes subspace models at high resolution. More advanced techniques use nonlinear manifold embedding^6,28–31^ or deep generative models^7,8^ to construct a nonlinear manifold in the space of 3D density. Density-based methods, such as 3DVA and cryoDRGN, capture conformational and compositional heterogeneity, but they do not model protein motion or the preservation of local geometry. As such, they do not enable (nonrigid) alignment and the aggregation of signal across the conformational landscape.

Methods for continuous heterogeneity with models of motion have begun to emerge. Hypermolecules^32^ represent heterogeneous protein density in a higher-dimensional space, which have the potential to capture nonrigid motion and structure but this has yet to be demonstrated on experimental data. e2gmm (ref. ^9^) represents a density using a Gaussian mixture model. Changes in 3D density are inferred by a neural network that adjusts the positions and amplitudes of the Gaussian components. Motion is estimated only at the Gaussian centers, in contrast to a dense deformation field with which one can align the entire density map for different conformations. These design choices and other limitations mentioned by Chen and Ludtke^9^ constrain model resolution, and a method to improve reconstruction quality of the aggregate density beyond the initial rigid consensus reconstruction is not proposed.

3DFlex recovers nonrigid motion from single-particle cryo-EM data of large complexes and smaller membrane proteins. The learned motion allows 3DFlex to improve resolution and map quality of flexible regions in the canonical density map by aggregating structural information across a protein’s conformational landscape. While these capabilities can help shed light on biological function, the current method has limitations which highlight interesting directions for future research.

One limitation is that 3DFlex was not designed to handle compositional heterogeneity, where density appears and disappears between discrete states. In these cases, 3DFlex may use its model capacity to approximate disappearance as spatial diffusion of density rather than capturing flexibility. Currently, we recommend 3DFlex be run on particle subsets with little compositional heterogeneity. The deformation model of 3DFlex is, however, expected to find deformations between discrete conformational states, aligning them to the canonical map. Note that in the absence of particle data in intermediate conformations, 3DFlex is guided only by its inductive bias and rigidity priors in modeling these transitions.

In our results on the translocating ribosome, 3DFlex found large, functionally relevant motions. Molecular machines such as the ribosome do undergo these motions during a reaction, but their function also relies on small, intricate changes, such as the motion of a single side chain or loop^15^. Some such changes remain beyond the reach of 3DFlex. Similarly, while 3DFlex improved the resolution of large regions of the ribosome compared to rigid refinement with all particles, similar or better resolution of smaller moving parts can be achieved by repeated application of focused 3D classification and masking^15^. One potential use of 3DFlex is to provide an interpretable latent representation of the conformational landscape, facilitating particle selection for conventional processing. Focused application of 3DFlex with masks may also be beneficial.

With the ability of methods such as 3DFlex to resolve motion, there is a need for methods to validate that the deformation fields are well supported by the image data. These may be statistical methods, such as FSC^33^, or perhaps fitting atomic models across the latent space to enable further validation.

Beyond the current architectural and optimization choices used in 3DFlex, alternatives may provide gains, one example being the use of neural fields^34–36^ instead of real-space voxel grids and the tetrahedral mesh encoding of motion. They can be optimized with gradient-based algorithms, but are not limited to spatially uniform resolution. Regularization of 3DFlex, both for the flow generator and the canonical density, can make use of structurally aware priors. For example, backbone models can be used to influence mesh construction and allow fine-grained structures to be resolved. The flow generator can also be expanded to allow for certain motions (for example, rotary) to be more naturally encoded, and the architecture can be expanded to handle compositional variability.

## Methods

3DFlex is a generative neural network method for determining, from cryo-EM particle images, the structure and motion of flexible protein molecules at atomic resolutions. In what follows, we outline the formulation of the model and the essential design choices of the model architecture and learning procedure. We also discuss hyper-parameter selection for effective use.

Central to 3DFlex is the overarching assumption that conformations of a dynamic protein are related to each other through deformation of a single 3D structure. Specifically, a flexible molecule is represented in terms of (1) a canonical 3D density map, (2) latent coordinate vectors that specify positions over the protein’s conformational landscape and (3) a flow generator that converts a latent coordinate vector into a deformation field that convects the canonical map into the corresponding protein conformation. The canonical 3D map, the parameters of the flow generator and a latent coordinate vector for each particle image are the model parameters that are initially unknown. They are jointly learned from experimental data.

Under the 3DFlex model (Fig. 1), a single-particle 2D image *I*_*i*_ is generated as follows. First, the *K*-dimensional latent coordinates **z**_*i*_ of the particle are input to the flow generator *f*_*θ*_(**z**_*i*_). The generator provides a 3D deformation field, denoted **u**_*i*_(**x**), where **x** is a 3D position and *θ* denotes the parameters of the generator. The deformation vector field and the canonical 3D density map *V* are input to a convection operator, denoted *D*(**u**_*i*_, *V*), which outputs a convected density, denoted *W*_*i*_. The 2D particle image *I*_*i*_ is then a CTF-corrupted projection of *W*_*i*_, plus additive noise *η*; that is,

2 $$\begin{array}{lll}{I}_{i}&=&{C}_{i}\,P({\phi }_{i})\,{W}_{i}\,+\,\eta \\ &=&{C}_{i}\,P({\phi }_{i})\,D({f}_{\theta }({{{{\bf{z}}}}}_{i}),V)\,+\,\eta .\end{array}$$

Here, *C*_*i*_ denotes the CTF operator and *P*(*ϕ*_*i*_) is the projection operator for pose *ϕ*_*i*_, specifying the rigid transformation between the microscope coordinate frame and the coordinate frame of the canonical map.

Fitting 3DFlex to experimental data entails optimizing the flow generator parameters *θ*, the canonical density map *V* and the per-particle latent coordinates **z**_1:*M*_, to maximize the likelihood of the experimental data under the probabilistic model (equation (2)). This is equivalent to minimizing the negative log likelihood,

3 $${E}_{{{{\rm{data}}}}}(V,\theta ,{{{{\bf{z}}}}}_{1:M})\,=\,\frac{1}{2}\,\mathop{\sum }\limits_{i=1}^{M}\,{\left\Vert {I}_{i}-{C}_{i}P({\phi }_{i})D\left({f}_{\theta }({{{{\bf{z}}}}}_{i}),V\right)\right\Vert }^{2},$$

where *M* is the number of particle images. Our current model assumes additive white noise, however extensions to colored noise are straightforward. We also assume that poses *ϕ*_*i*_ and CTF estimates are known, for example, from a standard cryo-EM refinement algorithm, although these parameters could also be reoptimized in the 3DFlex model.

The 3DFlex framework entails several important design choices that define the architecture of the 3DFlex model. Computationally determining structure and motion from noisy cryo-EM data is a challenging problem. As such, discussion of the design choices below provides insight into the working model, reflecting our exploration of different designs and hyper-parameter settings during the development of 3DFlex.

### Flow generator

We use a fully connected deep neural network (often called a multi-layer perceptron or MLP) with rectified linear activation functions for the flow generator. The input **z** is the low-dimensional latent coordinate vector for a given image, and the output is a 3D flow field **u**(**x**). The number of hidden units per layer (typically 32–128) and the number of layers (typically 2–8) are adjustable hyperparameters. The final layer is linear (without biases or nonlinear activation). The default 3DFlex architecture is a six-layer MLP with 64 units per hidden layer, which works well on a wide range of experimental datasets we have used. Larger models are more expressive but may also be more prone to overfitting. Accordingly, smaller networks are often useful for proteins with smooth motions, such as TRPV1 in the section TRPV1 ion channel: capturing flexible motion improves resolution.

### Latent coordinates

The latent space in 3DFlex represents the conformational landscape. Different latent positions correspond to different deformations of the canonical map. 3DFlex defaults to a 2D latent space but allows the user to explore other options. For more complex motions, a large latent space allows for discovery of multiple dimensions or types of motion. Typically, we use latent dimensions between 2 and 6; in our experience it is useful to start with two dimensions, and then incrementally increase the latent dimension to explore more complex models.

### Auto-decoder

A key step in training the 3DFlex model is to infer the latent coordinates (also known as the embedding) for each input particle image. In probabilistic terms, given an image *I* and the current estimate of the 3D map, one wants to infer the posterior distribution over latent coordinates **z**, where high probability coordinates are those for which the flow generator and canonical map explain the image well. Equivalently, these are the latent coordinates that yield low values of the negative log likelihood (equation (3)).

Determining the exact posterior distribution is, however, intractable for problems such as 3DFlex. So, instead, we turn to approximate inference. One approach, commonly used in variational auto-encoders (VAE)^37^, is amortized variational inference, in which a single feed-forward neural network (the encoder) is used to approximate the posterior for any image. Given an input image, the encoder outputs the mean and covariance over latent coordinates, essentially ‘inverting’ the generative model to predict the most likely latent coordinates for that image. This approach has been used by deep-learning based heterogeneity methods^7–9^. In the context of 3DFlex, the encoder would be trained jointly with the flow generator and the canonical map, to maximize the likelihood of the particle images.

VAEs are usually stable to train and inference is fast, requiring just a single pass through the encoder network. They also incorporate a prior over latent coordinates that helps to regularize the latent space, encouraging smoothness. Nevertheless, amortized inference can be problematic. Primarily, it can be difficult for the encoder network to accurately approximate the posterior defined by the generative model^38^. In the context of 3DFlex, an effective encoder network must be able to invert CTF corruption, 3D-to-2D projection, and 3D deformation to infer a latent state given a noisy 2D image. Motion must be learned in tandem with the generative model, so when the flow generator shifts a particular subunit up or down, the encoder must simultaneously learn the same motion and how it appears from 2D viewing directions to infer the position of the subunit in an image. This learning task is difficult, especially given the high noise levels in experimental images. Indeed, we did not find amortized inference effective for resolving high-resolution structure and motion.

In 3DFlex, we instead adopt an auto-decoder model, where we perform inference by optimizing a point estimate of the latent coordinates independently for each image, taking advantage of the structure of the generative model directly. Although more computationally expensive than amortized inference with an encoder network, this direct inference is more precise. This allows 3DFlex to capture structure and motion with sufficient detail to resolve flexible protein regions to higher resolution than is possible with previous methods.

The generative model for 3DFlex is end-to-end differentiable, and so one can compute gradients of the data likelihood with respect to the latent coordinates for each image, and then use gradient-based optimization to perform inference. When the dimensionality *K* of the latent space is small enough, it is also possible to use coordinate descent. We found the latter approach to be simpler and equally effective in our experiments.

### Noise injection and prior on latents

Instead of computing a point estimate for **z** given *I*, one could explicitly approximate the posterior *p*(**z** ∣ *I*). In doing so, one captures uncertainty in **z** that can be used to help regularize the model and encourage smoothness of the latent space; that is, ensuring that nearby latent coordinates yield similar deformation fields, and hence similar conformations. In 3DFlex, we find that directly adding noise to the point estimate during training produces a similar positive effect. This method can be likened to variational inference with a Gaussian variational family with a fixed covariance, and has been used to regularize deterministic auto-encoders^39^. Finally, in addition to noise injection, we use a Gaussian prior on latent coordinates with unit variance to help control the spread of the latent embeddings for different particles within a given dataset, and to center the distribution of latent embeddings at the origin in the latent space.

### Real versus Fourier space

Algorithms for single-particle reconstruction commonly represent 3D maps and 2D images in the Fourier domain. Working in the Fourier domain reduces the computational cost of CTF modulation and image projection (via the Fourier-slice theorem). It also allows maximum-likelihood 3D reconstruction with known poses in closed-form (for example, see refs. ^40,41^). On the other hand, the convection of density between conformations is more naturally expressed in real space, where structures in the canonical density map *V* need to be shifted, rotated and potentially deformed to produce densities consistent with the observed particles.

In 3DFlex, we represent the canonical density *V* in real space, as a voxel array of size *N*^3^. Convection and projection are performed in real space, and in practice are combined into a single operator that does not store *W*_*i*_ explicitly. Once the projected image of the convected map is formed, it is transformed to Fourier space and CTF modulated, and transformed back to real space to be used with the observed image for likelihood computation. Computationally, real-space convection and projection are far more expensive than Fourier-space slicing, and the fast Fourier transform for CTF modulation must be applied for every image in the forward pass, and also in the backward pass for computing gradients. Nevertheless, we find that in 3DFlex, high-resolution 3D reconstruction of the canonical map is possible in real space when using suitable optimization techniques (below).

### Convection operator

Convection of density is an essential element of 3DFlex, modeling the physical nature of protein motion, thereby allowing high-resolution structural detail from experimental data to backpropagate through the model. There are several ways to construct a convection operator. One is to express the flow field as a mapping from convected coordinates (that is, voxels in *W*_*i*_) to canonical coordinates. Convection then requires interpolating the canonical density *V* at positions specified by the flow field. For density conservation, the interpolated density must be modulated by the determinant of the Jacobian of the mapping, which is challenging to compute and differentiate. Instead, the flow in 3DFlex, **u**_*i*_, is a forward mapping from canonical coordinates in *V* to the deformed coordinates in *W*_*i*_. This approach naturally conserves density, as every voxel in *V* has a destination in *W*_*i*_ where its contribution is accumulated through an interpolant function. The convected density at location **x** can be written as

4 $${W}_{i}({{{\bf{x}}}})\,=\,\mathop{\sum}\limits_{{{{\bf{y}}}}}k(\,{{{\bf{x}}}}-{{{{\bf{u}}}}}_{i}({{{\bf{y}}}})\,)\,V({{{\bf{y}}}})$$

where **u**_*i*_ = *f*_*θ*_(**z**_*i*_), *k* is an interpolation kernel with finite support and the summation is over 3D spatial positions **y** of the canonical map. Here, divergence and convergence of the flow field must be treated carefully to avoid undesirable artifacts such as holes, Moiré patterns and discontinuities. We found high-order (for example, tricubic) interpolation and strong regularization (below) useful to ensure accurate interpolation and artifact-free gradients.

### Regularization via tetrahedral mesh

As one adds capacity to a model such as 3DFlex, the propensity for overfitting becomes problematic without well-designed regularization. In early formulations of 3DFlex, overfitting resulted in the formation of localized, high-density points (‘blips’) in the canonical map, along with flow fields that translated these aberrations by large distances to explain noise in the experimental images. This problem was especially pronounced with smaller proteins, higher levels of image noise and membrane proteins containing disordered micelle or nanodisc regions (that is, structured noise). Overfitting also occurs when the regularization is not strong enough to force the model to separate structure from motion. For example, rather than improve the canonical density with structure common to all conformations, the model sometimes learned to deform a low-resolution canonical density to create high-resolution structure (with highly variable local deformations).

To address these issues, 3DFlex exploits previous knowledge of smoothness and local rigidity in the deformation field. In particular, it is unlikely that natural deformations would involve large discontinuities in regions of high density; for example, an α-helix should not be sheared into disjoint pieces. It is also unlikely that deformations will be highly nonrigid at fine scales in regions of high density; at the extreme, bond lengths should not stretch or compress substantially. With these intuitions, we tried simple regularizers acting on flow fields defined at each voxel, such as limiting the frequency content of the flow field or penalizing its curvature. However, these regularizers were difficult to tune and did not prevent overfitting reliably.

3DFlex instead models flow generation using finite-element methods. A tetrahedral mesh covering regions of high density is generated in the canonical frame, based on a preliminary consensus refinement or input by the user (Mesh generation and customization below). The deformation field is parameterized by a 3D flow vector at each vertex of the tetrahedral mesh. The deformation field is then interpolated using linear finite-element method shape functions within each mesh element. Smoothness is a function of the size of mesh elements (an adjustable parameter) and is enforced implicitly through interpolation and the fact that adjacent elements can share vertices.

We also encourage local rigidity of the flow in each mesh element. The deformation field within the *j*th tetrahedral element for image *i*, denoted **u**_*ij*_(**x**) can be written as a linear mapping:

5 $${{{{\bf{u}}}}}_{ij}({{{\bf{x}}}})\,=\,{A}_{ij}{{{\bf{x}}}}+{{\bf{b}}}_{ij}$$

where matrix *A* and vector **b** are uniquely determined from 3D flow vectors at the element vertices. We quantify local nonrigidity in terms of the distance between *A* and the nearest orthogonal matrix (in a mean squared-error sense^42,43^). In particular, we measure the squared deviation of the singular values of *A* from unity. Letting ${s}_{ij}^{\ell }$ be the *ℓ*th singular value of *A*_*ij*_, we express the local rigidity regularization loss as

6 $${E}_{{{{\rm{rigid}}}}}\,=\,\mathop{\sum}\limits_{i}\mathop{\sum}\limits_{j}{w}_{j}\mathop{\sum }\limits_{\ell =1}^{3}{({s}_{ij}^{\ell }-1)}^{2}$$

where *w*_*j*_ are weights defining the strength of the prior within each mesh element, based on the density present within the *j*th mesh element. The densest elements have weight 1.0 and empty elements have a lower weight, by default 0.5. This weighting ensures that deformation fields are encouraged to compress and expand empty space around the protein. Weight customization is possible but not necessary; the default weights were sufficient for all of our experiments.

### Mesh generation and customization

3DFlex can take as input any mesh construction and topology. The size and connectedness of the mesh become hyperparameters of the model and provide inductive bias in estimating deformations. By default, 3DFlex automatically generates a regular mesh of tetrahedral elements of a fixed size and spacing made to cover the density of the input consensus refinement, where all elements share vertices with their direct neighbors.

One can also create an customized mesh by modifying an automatically generated mesh, for example with variable sized mesh elements, overlapping elements or where it is not necessary that neighboring elements share vertices. The last case can be thought of as introducing ‘cuts’ into the mesh at the faces where neighboring elements do not share vertices. At these faces, the deformation model has the freedom to move adjacent mesh elements in opposite or shearing directions.

Extended Data Fig. 1 shows an example of cutting a mesh to allow nearby domains of the SARS-CoV-2 spike protein to move independently under the 3DFlex model. A regular mesh (Extended Data Fig. 1a) may contain mesh elements that overlap adjacent domains of the protein, making the model unable to move one domain without affecting the motion of the other. Instead, if the subdomains can be separated (for example, as in Extended Data Fig. 1b) then each subdomain can be covered with a submesh. The submeshes can be fused together only at the interfaces where the density is continuous. In the example of the spike protein (Extended Data Fig. 1c), these fusions could be made between the lower S2 trimer (orange) and each of the NTD and RBD domains in S1 (purple, blue, pink, yellow, orange, green). 3DFlex includes a mesh utility to help simplify the construction of such fused meshes. A user can start with a consensus reconstruction map, and segment this at a coarse resolution into subdomains that are to be separated, using commonly available 3D segmentation tools (for example, ref. ^44^). These segments and the consensus map are input into the 3DFlex mesh utility. The utility generates a base regular tetrahedral mesh over the extent of the consensus map (Extended Data Fig. 1a). It also automatically expands each segment to include all voxels that are nearest to that segment, thereby defining coarse boundaries between subdomains (Extended Data Fig. 1b). A submesh is created for each subdomain using vertices from the base mesh that cover the boundaries (Extended Data Fig. 1c). The utility then fuses the submeshes at continuous interfaces indicated by the user. Providing 3DFlex with a custom mesh topology provides additional inductive bias, allowing it to better represent physically plausible deformations, but it does not introduce additional information about the directions or magnitudes of motion at any of the mesh nodes, as all deformations are still learned from the data during training.

Whether using a regular or custom mesh, there is substantial latitude in specifying the mesh. Where motions are smooth, the size and shape of mesh elements and their precise locations are not critical since they only serve to ensure the deformation is smooth, and the flow generator is able to displace the mesh elements (including changing their size or shape) during deformation. Likewise for custom meshes, the separation of subdomains does not need to be ‘exact’ as the canonical voxel density values and structure within each region of the mesh are still learned from the data by 3DFlex.

### Optimization of flow and structure

3DFlex is end-to-end differentiable, allowing gradient-based optimization to be used to train the flow generator and learn the canonical density that best explains the data. We use either Adam^45^ or stochastic gradient descent with Nesterov acceleration^46^ with minibatches of at least 500 images, due to the high levels of image noise. Inference of the latent coordinates for each image in a minibatch is performed before computing gradients with respect to the canonical density and flow parameters. The loss function (equation (7)) is a weighted sum of the data log likelihood (equation (3)) and the nonrigidity penalty (equation (6)):

7 $$L\,=\,{E}_{{{{\rm{data}}}}}+\lambda \,{E}_{{{{\rm{rigid}}}}}.$$

The regularization hyper-parameter *λ* is set to 2.0 by default. We find that with small adjustments, often in the range 0.5 and 5.0, one can modify the degree of nonrigidity in the regularizer, often yielding improved models.

During optimization, we use frequency marching, learning the model in a coarse-to-fine manner. The canonical density *V* is constrained to be low-pass, with an upper frequency band-limit that increases over iterations. The frequency and learning rate schedule, and *λ*_rigid_, can be tuned for each dataset in our current implementation, but default values work well in most cases. Optimization is done with a box size, *N* = *N*_*L*_, that is typically smaller than the raw size of the particle images *N*_H_. In this way, 3DFlex optimization only has access to relatively low frequencies from the observed images (that is, below the Nyquist frequency for the smaller box size). This is useful as it makes optimization faster, it allows one to use the resulting alignment of higher frequencies as a way to validate the quality of the deformation and makes it easier to rule out overfitting.

To initialize training, we set the canonical density *V* to be a low-pass filtered version of a consensus nonuniform refinement map in cryoSPARC^17^, given the same particle images. The parameters of the flow generator are randomly initialized. The latent coordinates are initialized to zero by default. Optionally, one can also use different initial values. With smaller, low signal-to-noise particles, such as TRPV1 for example, we find that initializing with latent coordinates from 3DVA (ref. ^5^) in cryoSPARC^18^ improves results.

We find that simultaneously training the canonical density *V* and flow generator parameters *θ* leads to overfitting after thousands of gradient iterations, despite strong regularization. However, we find that a form of block coordinate descent provides a stable way to optimize 3DFlex. By default, when initializing from zero for latent coordinates, we first perform five epochs of optimizing *θ* and latent coordinates with *V* fixed. When initializing from input latent coordinates, we perform the first five epochs with the latent coordinates fixed. Then, we proceed to epochs alternating between optimization of *V* and optimization of *θ* and latent coordinates, repeating until convergence.

### High-resolution refinement and validation

With the ability of 3DFlex to capture motion and latent coordinates of each particle image, it becomes possible in principle to attempt recovery of high-resolution detail in flexible parts of protein molecules that would otherwise be blurred using conventional refinement methods.

3DFlex is optimized at a small box size, *N* = *N*_*L*_. Once optimization has converged, we freeze the flow generator parameters *θ* and the latent coordinates ${{{{{\bf{z}}}}}}_{1:M}$, and then transfer them to a new model at full resolution, with *N* = *N*_H_. We partition the particles using the same split that was used in the consensus refinement (from which we obtained the poses {*ϕ*_*i*_}). For each half-set we initialize the canonical density *V* to zero, and re-optimize it at full box size *N*_H_, with the other parts of the model fixed. In the same way as established reconstruction validation methods^20,33^, the two resulting half-maps can be compared via FSC. Correlation beyond the training-time Nyquist resolution limit indicates that consistent signal was recovered from both separate particle sets, as opposed to spurious correlation or overfitting of the model. This correlation serves to validate the improvement in reconstruction of flexible regions with the learned deformation model.

To this end, we need to optimize *V* at high resolution under the full 3DFlex model for each half-set. We initially experimented with localized reconstructions (in Fourier space) but encountered issues with nonrigidity and curvature. We also found that minibatch stochastic gradient descent methods for directly optimizing *V* did not yield high quality results, potentially because noise in minibatch gradient estimates is problematic for this task. Instead, we were able to solve the problem using full-batch L-BFGS (ref. ^16^) in real space. This approach is substantially more expensive than Fourier-space reconstruction, and requires many iterative passes over the whole dataset. However, it is notable in that it allows 3DFlex to solve high-resolution detail in all flexible parts of the protein simultaneously, without making explicit assumptions of local rigidity or smoothness.

### Symmetry

Symmetric particles will not necessarily maintain symmetry during conformational changes, since the motion of individual subunits or the overall structure can break symmetry. In the current method, we do not enforce symmetry during training of 3DFlex. However, in processing of TRPV1 we do assume C4 symmetry in the initial rigid consensus refinement. To facilitate comparison with rigid reconstruction baselines where symmetry was enforced, we can apply symmetrization to the reconstructed half-maps after high-resolution reconstruction under 3DFlex. This makes the assumption that the canonical density of the molecule is indeed symmetric, and it is only the conformational changes that break symmetry. The symmetrized half-maps can then be compared using gold-standard FSC with half-maps from rigid reconstruction where symmetry was enforced, as we showed for the TRPV1 dataset in Fig. 3. Likewise for TRPV1, we are able to compare the symmetrized 3DFlex reconstructions with rigid local refinement by first applying C4 symmetry expansion to the particle images, and then performing local refinement within a mask that only includes the region of interest in one asymmetric unit of the molecule (Fig. 3h). In this way, we can ensure that the same number of asymmetric units were seen in rigid, local and 3DFlex refinements.

### Reporting summary

Further information on research design is available in the Nature Portfolio Reporting Summary linked to this article.

## Online content

Any methods, additional references, Nature Portfolio reporting summaries, source data, extended data, supplementary information, acknowledgements, peer review information; details of author contributions and competing interests; and statements of data and code availability are available at 10.1038/s41592-023-01853-8.

## Supplementary information

Supplementary Information Supplementary Note, Figs. 1 and 2 and Table 1.

Reporting Summary

Supplementary Video 1 Video of 3DFlex results for snRNP.

Supplementary Video 2 Video of 3DFlex results for TRPV1 (motion).

Supplementary Video 3 Video of 3DFlex results for TRPV1 (reconstruction).

Supplementary Video 4 Video of 3DFlex results for SARS-CoV-2 spike.

Supplementary Video 5 Video of 3DFlex results for Integrin.

Supplementary Video 6 Video of 3DFlex results for Ribosome.

## Data Availability

Publicly available datasets EMPIAR-10073 (tri-snRNP spliceosome complex), EMPIAR-10059 (TRPV1 ion channel), EMPIAR-10516 (SARS-CoV-2 spike protein), EMPIAR-10345 (*α**V**β*8 integrin), EMPIAR-10792 (translocating ribosome) and EMPIAR-10025 (T20S Proteasome) were used in this study.

## Code availability

3DFlex is implemented within the CryoSPARC software package. CryoSPARC is available in executable form free of charge for nonprofit academic research use at www.cryosparc.com.
