# Supplementary material for: 3DFlex: determining structure and motion of flexible proteins from cryo-EM
Source: Nat Methods. 2023 May 11;20(6):860–70. doi: 10.1038/s41592-023-01853-8 (PMC10250194; doi:10.1038/s41592-023-01853-8)
Supplement: Supplementary file 1 — Supplementary Note, Figs. 1 and 2 and Table 1. [file 41592_2023_1853_MOESM1_ESM.pdf]

nature methods

Article

https://doi.org/10.1038/s41592-023-01853-8

3DFlex: determining structure and motion of flexible proteins from cryo-EM

In the format provided by the authors and unedited

Supplementary Information for

3DFlex: Determining Structure and Motion of Flexible Proteins from Cryo-EM

The follow sections provide supplementary material concerning the quantitative validation of 3DFlex, first with synthetic data for which ground truth is available, and second, with a T20S Proteasome dataset to show that 3Flex does not yield poorer quality map structure compared to conventional refinement when applied to data with no substantial flexibility. Finally, we provide a table that summarizes the data and model parameters used in each of the experimental datasets in the main body of the paper.

Validation of 3DFlex

To help validate 3DFlex we generated a moderately sized synthetic dataset with prescribed deformations. This allows us to compare 3DFlex models against ground truth. We also consider data in which there is little or no conformational variation, which helps to validate that 3DFlex does not harm resolution of map quality on data from rigid particles.

We use a density map of the T20S Proteasome (PDB 6BDF) at 2.5Å resolution, and define a ground-truth deformation that bends and twists the 3D map (Supplementary Figure 1a), to generate 100,000 synthetic particle images at random orientations with latent coordinates drawn from a Gaussian (Supplementary Figure 1b). The particle images, with box size 384 and pixel size 0.87Å, are individually CTF corrupted covering a range of defocus values, and realistic noise with signal-to-noise-ratio $1/200$ is added (Supplementary Figure 1c). Conventional reconstruction of the particles from their ground-truth alignments yields a poor density map with substantial loss of resolution in the moving regions (Supplementary Figure 1h).

Beyond the use of ground truth alignments for the consensus reconstruction, the synthetic data are processed the same way as experimental data in the main body of the paper. The particle images are downsampled to a box size of 128 (pixel size 2.61Å) for training 3DFlex. We use two latent dimensions, matching the degrees of freedom in the synthetic bending and twisting. The tetrahedral mesh was automatically generated to cover the density map from the input 3D reconstruction, with 1665 vertices and 6389 cells, each about 13Å wide. Total training time for 3DFlex is 10 hours.

3DFlex recovers a high-resolution density map, the deformation fields, and the latent coordinates of each particle image. The flow generator captures the full range of deformations present in the data and recovers the true latent structure to within an arbitrary non-linear transformation (indicated by corresponding red grid lines in Supplementary Figure 1b). We measure the precision of the learned deformation model by computing, at each voxel, the RMS error between learned and ground-truth deformation across all particles in the dataset. While ground truth displacements reach $\pm$9Å, 3DFlex achieves an average RMS error of 0.4Å, with all voxels having errors less than 1.0Å (Supplementary Figure 1d,e). The precision of the estimated deformation field allows 3DFlex to recover the high-resolution density even in moving regions at the top of the structure (Supplementary Figure 1h). Global FSC resolution compared to GT improves from 3.4Å to 2.6Å (Supplementary Figure 1f), and locally in the top quarter of the map where motion is largest, from 6.0Å to 2.7Å (Supplementary Figure 1g). The GT density is limited to 2.5Å so these results indicate that 3DFlex recovered nearly all the available signal.

Finally, we also applied 3DFlex to an experimental dataset of 10,000 particle images of the T20S Proteasome (EMPIAR-10025) for which we did not expect significant conformational heterogeneity. We used default parameters, a mesh of cell size 14Åwith 916 vertices and 3288 cells and $K = 2$ latent dimensions. 3DFlex learned that

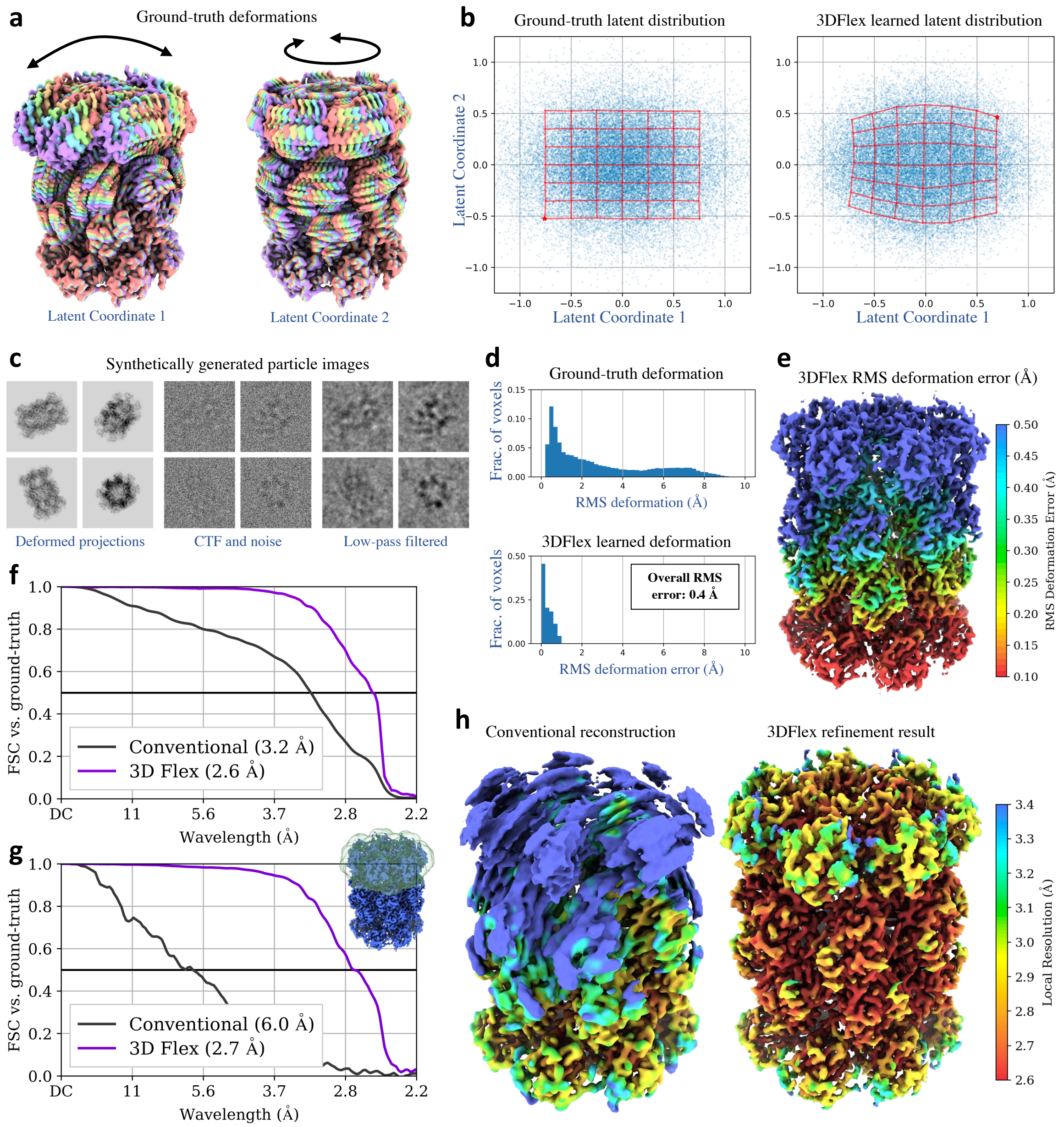

Supplementary Figure 1 3DFlex is applied to (synthetic) particle images of a T20S Proteasome that bends and twists. **a**: Ground truth (GT) density map at 2.5Å and deformation fields. **b**: Distributions of 2D latent coordinates. Exemplar points (red) show correspondence between GT and learned latent spaces. **c**: Example particle images (low-pass filtered only for visualization). **d**: (top) Histogram of GT RMS deformation at each voxel (averaged over particles), showing the fraction of protein voxels with a given average deformation magnitude. (bottom) Histogram of RMS error in learned deformation model. **e**: Estimated canonical map, colored by error in learned deformations. **f**: Global FSC between the canonical density and GT density. **g**: Local FSC to GT within a mask covering the map region with the largest motion. **h**: Conventional (rigid) and 3DFlex reconstructions colored by local resolution value.

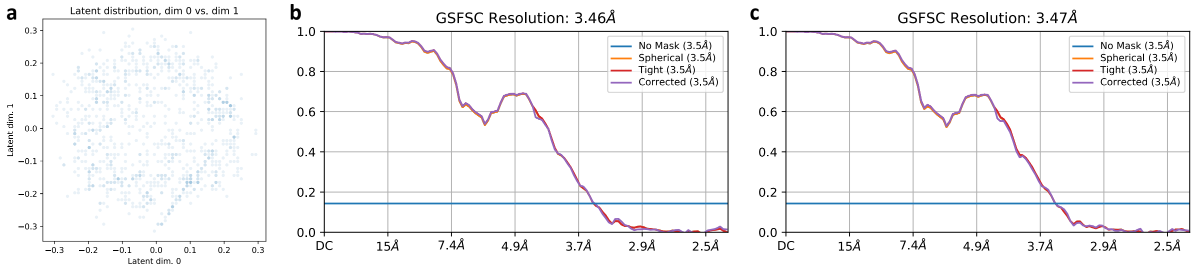

Supplementary Figure 2 Results on T20S particle images showing that the resolution of a rigid molecule is not improved or degraded by 3DFlex. **a**: Distribution of latent coordinates **b**: FSC curves for conventional homogeneous reconstruction **c**: FSC curves for 3DFlex refinement of the canonical density.

near-zero deformation best fit the data. As shown in Supplementary Figure 2a, the per-particle latent coordinates are centered about the origin in the latent space, with some spread, as the model includes a prior that latent coordinates should be distributed as a standard normal distribution. Nevertheless, the flow generator encodes that there is no deformation of the particle, regardless of the position in the latent space. As such, there is no substantial change in the resolution or quality of the density map compared to conventional refinement. Supplementary Figures 2b,c show the gold-standard FSC curves for a conventional homogeneous reconstruction and that obtained from the high-resolution reconstruction of the canonical density in 3DFlex (bottom), the resolutions of which are 3.46Å and 3.47Å respectively. Experiments on other datasets similarly show that rigid parts of the proteins are not degraded (or improved) during optimization of 3DFlex, in part due to the regularizer on local rigidity.

3DFlex Parameters for Experimental Datasets

For reference, in Supplementary Table 1 we provide the 3DFlex model parameters and attributes that were used to process each of the five experimental datasets described in the main body of the paper. This includes resolution of the full resolution and down-sampled particle images, as well as the parameter values for the latent space, the neural network flow generator, the tetrahedaral mesh, and the mesh regularization constant. A discussion of the specific parameter choices, and the ways in which they affect the behavior of 3DFlex, can be found in Results and Methods.

	**Splicesome**	**TRPV1**	**Spike**	**Integrin**	**Ribosome**
# particle images	138,899	200,000	113,511	84,266	58,433
original box size	380	224	256	300	288
original pixel size	1.4Å	1.21Å	1.396Å	1.345Å	1.16Å
reduced box size	180	128	140	128	140
reduced pixel size	2.95Å	2.15Å	2.55Å	3.15Å	2.38Å
latent dimension	5	2	3	2	2
latent initialization	random	3DVA	random	random	random
# MLP layers	6	3	6	6	6
# hidden units / layer	64	32	64	64	64
# mesh vertices	1601	1054	1306	477	3700
# mesh cells	5859	3892	4353	1452	13475
approx. cell width	18Å	14Å	14Å	22Å	14Å
regularization λ (Eq. 7)	2.0	3.0	0.5	2.0	4.0
custom mesh	No	No	Yes	No	Yes
processing time (hours)	18	24	6	4	5

Supplementary Table 1 Table of attributes and parameters for each dataset used to test 3DFlex.
